# Predictive Value of Preoperative *HIF1A* and *EPAS1* Expression Levels and Inflammatory Response Molecules for Assessing the Risk of Complications in Cardiac Surgery Patients

**DOI:** 10.3390/ijms27156616

**Published:** 2026-07-24

**Authors:** Maria Kirillova, Dzhuliia Dzhalilova, Natalia Zolotova, Marina Diatroptova, Nikolai Fokichev, Maxim Babaev, Oksana Grin, Alexander Eremenko, Eduard Charchyan, Olga Makarova

**Affiliations:** 1 Petrovsky National Research Centre of Surgery, Moscow 117418, Russia; marusyasilina99@yandex.ru (M.K.); natashazltv@gmail.com (N.Z.); diatrop@inbox.ru (M.D.); maxbabaev@mail.ru (M.B.); grin_oksana@mail.ru (O.G.); aeremenko54@mail.ru (A.E.); charchmed@yahoo.com (E.C.);; 2 Faculty of Biology and Biotechnology, National Research University Higher School of Economics, Moscow 117418, Russia

**Keywords:** cardiac surgery, predictors, complications, hypoxia-inducible factor

## Abstract

Existing diagnostic markers of sepsis indicate already developed inflammatory complications, whereas predictors enable preoperative identification of high-risk patients for preventive measures. This study aimed to evaluate the prognostic value of preoperative inflammatory biomarkers for postoperative complications in cardiac surgery patients. Preoperatively, we assessed age, body mass index, EuroSCORE II (European System for Cardiac Operative Risk Evaluation II), complete blood count with hematological indices including neutrophil-to-lymphocyte ratio (NLR), serum cytokines, *HIF1A* (hypoxia-inducible factor 1-alpha), and *EPAS1* (endothelial PAS domain protein 1) expression in peripheral blood leukocytes. During surgery, cardiopulmonary bypass time, myocardial ischemia time, and blood loss were recorded. Postoperatively, complications, intensive care unit (ICU) stay, and total hospitalization were documented. No differences were found in non-modifiable risk factors or perioperative parameters between groups, except for longer ICU stay in the complications group. Preoperatively, absolute neutrophil count and NLR were higher in patients without complications, while eosinophil count, *HIF1A* expression, and *HIF1A*/*EPAS1* ratio were higher in those with it. The *HIF1A*/*EPAS1* ratio demonstrated the best diagnostic characteristics. Thus, preoperative predictors of postoperative inflammatory complications include low neutrophil count and NLR, as well as high *HIF1A* expression and *HIF1A*/*EPAS1* ratio. These markers may facilitate early risk stratification and preventive strategies before surgery.

## 1. Introduction

Cardiac surgery is actively developing due to new technologies, as well as modern diagnostic and therapeutic approaches to cardiovascular diseases. Nevertheless, the infectious and inflammatory complications ratio after cardiac surgery remains high, ranging from 4.9 to 30.8%, and 30-day mortality—from 1 to 4% [1,2].

Unlike diagnostic markers that indicate already developed inflammatory complications, predictors allow high-risk patients identification before surgical intervention and preventive measures—preoperative preparation optimization, surgical tactics determination, and targeted postoperative monitoring. Existing markers, which include about 1000 molecules, among which procalcitonin, C-reactive protein, endotoxin, presepsin, and others are most frequently identified, have limited predictive value [3,4]. Furthermore, hematological indices such as the neutrophil-to-lymphocyte ratio (NLR) can serve as markers for postoperative period severity assessment. In reconstructive aortic surgery, the NLR index was higher in the complications group throughout the entire postoperative period (6 days after surgery) [5]. Moreover, when this index exceeds 7.8 on the 3rd ICU (Intensive Care Unit) day, the inflammatory complications risk can be predicted with 72.7% sensitivity and 68.6% specificity [6]. However, most researchers evaluate various systemic inflammatory response biomarkers already after surgical intervention; therefore, the urgent task is predictors search that allow determining the inflammatory complications risk at the preoperative stage.

The main inflammatory response regulator is NF-κB, which activates proinflammatory cytokines and HIF-1α (Hypoxia-Inducible Factor), modulating the hypoxia response [7,8,9]. At the same time, inflammatory processes severity is influenced by sex, age, concomitant diseases, and individual hypoxia tolerance [10,11,12,13]. Previously, it was demonstrated that patients with high serum HIF-1α and VNN1 (vanin-1—pantetheinase, which hydrolyzes cysteamine, regulating inflammation and oxidative stress [14,15]) levels before surgery, as well as 24 and 72 h after it, and a high *HIF1A* expression level in leukocytes 72 h after surgical intervention had a higher postoperative infectious and inflammatory complications development risk [16], indicating the potentially high predictive value of molecules regulating the hypoxia response.

This study aimed to characterize the predictive value of molecules regulating cellular hypoxia and inflammation response preoperative levels for complications risk assessment in cardiac surgery patients.

## 2. Results

### 2.1. Patients’ Clinical Characteristics

**Figure 1:**
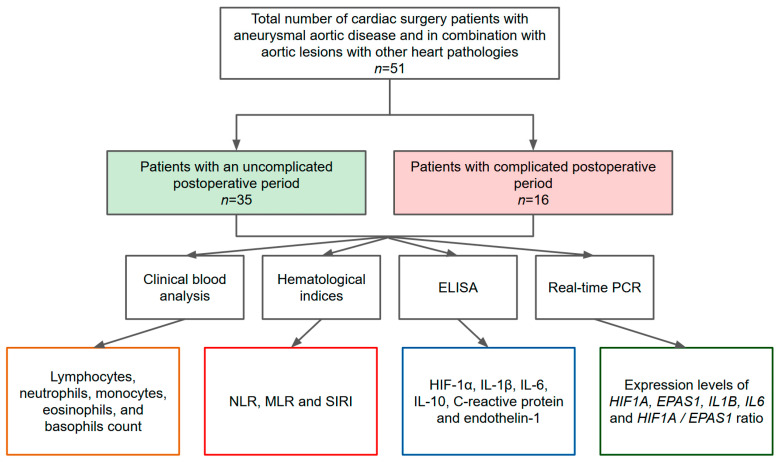
Scheme of the patients’ recruitment in a study, the tests performed and the analytes evaluated.

The included patients had a low EuroSCORE II mortality risk (3.3 (2.4–4.4)%), short cardiopulmonary bypass (CPB) duration (114.5 (89.5–150.8) min) and myocardial ischemia time (MIT, 88 (61.8–101.0) min), and a small intraoperative (700 (600–1000) mL) and total blood loss (1050 (900–1320) mL) volume. Local inflammatory complications in the postoperative period were identified in 16 patients (31%). Of these, thirteen were diagnosed with nosocomial pneumonia, accompanied in eight patients by respiratory failure. Three patients showed wound infection signs; vacuum aspiration therapy was used in two cases and surgical inflammatory focus control was performed. No fatal outcomes were observed. In 35 operated patients (69%), the postoperative period proceeded without complications and the average hospitalization length was 10 days, including ICU time (Figure 1).

Non-modifiable complications risk factors, such as age, body mass index (BMI), and EuroSCORE II adverse outcomes risk comparison revealed no statistically significant differences. Perioperative parameters—CPB, MIT, total and intraoperative blood loss—also did not differ. ICU time was significantly greater in the complications group (Table 1).

### 2.2. Analysis of Preoperative Parameters

**Figure 2:**
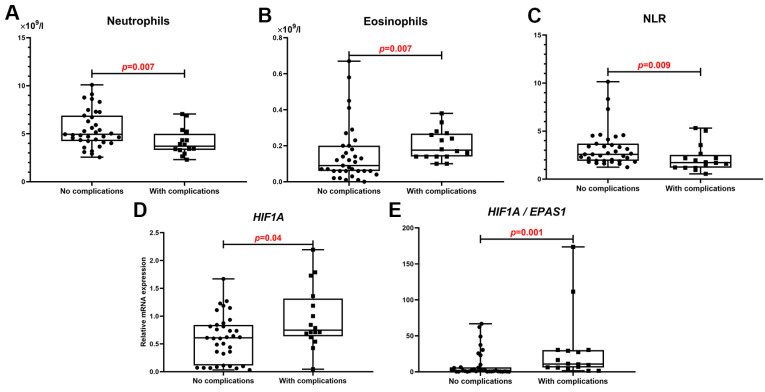
Postoperative complications predictors in cardiac surgery patients. (**A**): Neutrophils; (**B**): Eosinophils; (**C**): NLR; (**D**): *HIF1A* expression level; (**E**): *HIF1A* to *EPAS1* ratio. Me (25–75%), *p*—statistical significance of differences, Mann–Whitney test.

In the preoperative period, absolute neutrophil count and NLR index were higher in patients without complications after surgery, while eosinophil count, *HIF1A* expression level, and *HIF1A*/*EPAS1* ratio in peripheral blood leukocytes were higher in patients with a complicated postoperative period (Figure 2).

Leukocyte count, absolute lymphocyte, basophil, monocyte counts, MLR and SIRI indices as well as serum HIF-1α, IL-1β, IL-6, IL-10, C-reactive protein, and endothelin-1, and *EPAS1, IL1B,* and *IL6* expression levels in peripheral blood leukocyte concentrations revealed no differences between patients without and with complications (Table 2).

### 2.3. Assessment of the Identified Predictors Diagnostic Characteristics

**Figure 3:**
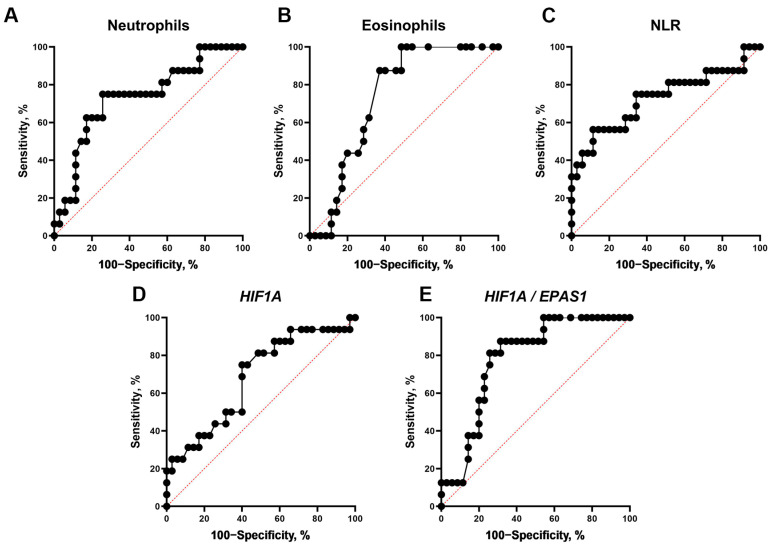
ROC-curves of postoperative complications predictors in cardiac surgery patients. (**A**): Neutrophils; (**B**): Eosinophils; (**C**): NLR; (**D**): *HIF1A* expression level; (**E**): *HIF1A* to *EPAS1* ratio.

The predictive value assessment of parameters determined before surgical intervention that differed between patients with complicated and uncomplicated postoperative courses demonstrated that the *HIF1A*/*EPAS1* expression ratio in peripheral blood leukocytes possesses the best diagnostic characteristics (sensitivity, specificity, likelihood ratio) (Figure 3, Table 3).

## 3. Discussion

Our study results indicate that the *HIF1A*/*EPAS1* ratio in peripheral blood leukocytes, determined before cardiac surgery, is an independent prognostic biomarker of postoperative inflammatory complications, possessing high sensitivity and specificity, and may be a useful biomarker for patients without traditional perioperative risk factors.

In cardiac surgery, the adverse outcomes risk assessment scale (EuroSCORE II) and traditional intraoperative risk factors (CPB and MIT duration, blood loss volume) cannot always explain the inflammatory postoperative complications development cause [17]. In our study, 30% of patients after combined reconstructive heart and major vessels surgeries had inflammatory complications, despite low preoperative risk (EuroSCORE II 3.26%), short MIT and CPB time, insignificant blood loss, and only 1-day ICU stay. This indicates hidden predictors’ existence associated with the individual inflammatory response, independent of anesthesiological and surgical intervention quality.

‘Classical’ inflammation markers content—leukocyte count, C-reactive protein concentration, IL-1β and IL-6 cytokines, as well as IL-10—was similar in both patient groups, indicating baseline inflammatory background and pro- and anti-inflammatory cytokines balance differences absence. The obtained results indicate these parameters low sensitivity and specificity as possible postoperative complications predictors, although their increase immediately after surgical intervention was demonstrated in many studies [18,19,20]. Moreover, when assessing the HIF-regulated pro-inflammatory cytokines *IL1B* and *IL6* expression levels, as well as serum endothelin-1 levels [21,22], we found no statistically significant differences between patients with complicated and uncomplicated postoperative courses. This may indicate that in our patient cohort, the systemic inflammatory response was not activated at the time of blood sampling. The observed *HIF1A*/*EPAS1* ratio differences likely reflect an individual predisposition to inflammatory complications rather than the current inflammatory status. This does not disprove the biological significance of the *HIF1A*/*EPAS1* ratio but rather underscores its prognostic value as a predictor detectable before clinical manifestations develop.

At the same time, neutrophil count and NLR in the preoperative period were statistically significantly higher in patients without complications after surgical intervention. In the Russian population, the peripheral blood absolute neutrophil count reference interval in men is 1.56–5.86 × 10^9^/L [23], indicating that despite the revealed differences, the parameter is within the normal range. At the same time, the NLR index in healthy individuals usually does not exceed 3.0 relative units [24]. According to the literature, elevated perioperative NLR is an independent early and late mortality predictor after cardiac surgery [25]. Furthermore, this index increased above 3 in the first three days after CPB surgeries correlates with hospital and ICU time [26]. However, in the described studies, neutrophil count and NLR index were determined in the peri- or postoperative period, while in our work these parameters were analyzed before surgical intervention. The low preoperative neutrophil count and NLR index value in comparison with patients without complications after surgery may indicate relative innate immunity immunosuppression in patients with a complicated postoperative period, which increases the inflammatory complications risk [27]. Our results are comparable with a similar study group investigation—an immunosuppressive phenotype in the preoperative period was identified in 35.7% of patients [28]. At the same time, the higher preoperative neutrophil content in patients without complications after surgical intervention may reflect an adequate functional organism reserve. Furthermore, the high preoperative eosinophil count in patients with a complicated postoperative period may reflect an initial immune response imbalance with Th2 type predominance. Eosinophils secrete a wide cytokine range: IL-2, IL-3, IL-4, IL-5, IL-6, IL-8, IL-10, IL-12, IL-13, IL-16, IL-18, TNF-α, IFN-γ, TGF-β, GM-CSF, and primarily realize Th2-type immune responses [29]. It was demonstrated that an elevated peripheral blood eosinophil count correlates with cardiovascular diseases risk [30]. In our study, this may also reflect a higher postoperative complications risk.

According to our data, the *HIF1A*/*EPAS1* expression ratio is characterized by high predictive value regarding complications risk after cardiac surgery. The transcription factors HIF-1α and HIF-2α (encoded by the *HIF1A* and *EPAS1* genes, respectively) are key cellular oxygen deficiency response regulators, whose transcriptional activity depends on hypoxia duration. Thus, the acute oxygen deficiency response is regulated by the HIF-1α isoform, while HIF-2α is activated during prolonged hypoxia [31,32]. Furthermore, these factors’ role in inflammation is quite well characterized in the literature. HIF-1α, through interaction with the main inflammatory response regulator NF-κB p65 subunit, which provides DNA binding, can induce proinflammatory cytokines *Il1b*, *Il6*, and *Tnfa* expression [22]. In macrophages, HIF-1α activation promotes their M1 phenotype polarization and proinflammatory cytokines production [33,34]. During surgical stress and ischemia-reperfusion, elevated HIF-1α content and transcriptional activity may contribute to a pronounced inflammatory response, oxidative stress, and tissue damage development [35]. At the same time, the HIF-2α isoform may have an anti-inflammatory effect. This protein target was demonstrated to be vascular endothelial protein tyrosine phosphatase, whose expression induction enhances cadherin dephosphorylation, increasing adherens junction integrity and endothelial cell barrier function [36]. HIF-2α protein also induces *Arg1* expression in macrophages, promoting their M2 phenotype polarization and anti-inflammatory cytokines synthesis [37]. Thus, a high *HIF1A*/*EPAS1* ratio value may be a hypoxic response imbalance sign and increased ischemia-reperfusion sensitivity cause, which, with equal CPB and MIT duration, leads to a more pronounced systemic inflammatory response after surgical intervention.

Our study has several limitations. It was performed on a small male patient group who did not have severe complications or fatal outcomes in the postoperative period. In addition, our study lacks a control group of healthy volunteers, which is necessary for future research and reference intervals evaluation. Furthermore, in this study, we did not evaluate endothelial permeability markers, such as soluble VE-cadherin, which could provide additional mechanistic insight into the postoperative complications development. Studying these markers could elucidate the mechanisms underlying susceptibility to inflammatory complications. Also, an important issue is the choice of a reference gene or a set of several housekeeping genes for real-time PCR, since it is known that their stability can be highly dependent on conditions and can be altered by stress factors or metabolic disturbances. On the other hand, this fact allowed us to reveal that the *HIF1A*/*EPAS1* ratio in peripheral blood leukocytes, determined before cardiac surgery, may be an independent postoperative inflammatory complications predictor, possessing high sensitivity and specificity, and may be a useful biomarker for patients without traditional perioperative risk factors. The organism’s hypoxic response balance and inflammatory reactions development relationship further study is required in a larger patient cohort with women and children inclusion, as well as to assess this parameter predictive value regarding severe complications and fatal outcomes.

Thus, we identified postoperative inflammatory complications predictors in cardiac surgery patients before surgical intervention—low absolute neutrophil count and NLR index, as well as high *HIF1A* expression level and *HIF1A*/*EPAS1* ratio in peripheral blood leukocytes, which will allow high-risk patients identification and preventive measures before surgery.

## 4. Materials and Methods

### 4.1. Patients

Human blood samples study permission was obtained from the Petrovsky National Research Centre of Surgery local ethics committee (Protocol No. 4 dated 20 April 2023). Written voluntary informed consent (File S1) and special patient check-list (File S2) was obtained from all patients in accordance with the Helsinki Declaration principles (as amended in 2013).

The inflammatory complications risk development prognosis was assessed in cardiac surgery patients with aneurysmal aortic disease and in combination with aortic lesions and other cardiac pathologies. All reconstructive aortic interventions were performed by one surgical team (Department of Reconstructive and Restorative Cardiovascular Surgery Head–Professor, Doctor of Medical Sciences, Corresponding Member of the Russian Academy of Sciences E.R. Charchyan) and anesthesiology team (Department of Anesthesiology and Resuscitation II Head–Professor of the Russian Academy of Sciences, Doctor of Medical Sciences B.A. Akselrod). ICU patient management protocols were also standard (ICU 2 Department Head–Doctor of Medical Sciences, Professor, Corresponding Member of the Russian Academy of Sciences A.A. Eremenko).

The study included 51 male patients aged 20 to 72 years. Inclusion criteria: male sex, age 18 to 75 years, aortic reconstructive surgical intervention performed by one surgical and anesthesiology team, patient’s voluntary informed consent presence. Exclusion criteria: female sex, age under 18 or over 75 years, other cardiac surgeries performed, reconstructive aortic surgeries performed by another surgical team, patient’s participation refusal, consciousness disorders preventing voluntary informed consent obtaining. The present study included only male patients, as inflammatory diseases are known to be more severe in males than in females [38,39], making the male population more appropriate for identifying predictors of postoperative inflammatory complications.

In the preoperative period, patient age, BMI, and EuroSCORE II adverse outcomes risk were assessed. During surgery, CPB duration, MIT, intraoperative and total blood loss (total volume: intraoperative plus the drainage reservoir volume during the first day) were assessed, and after surgical intervention, complications presence, ICU time, cardiac surgery department stay length, and total hospitalization time. The postoperative period was considered complicated if patients had local inflammatory processes (tracheobronchitis, pleurisy, pneumonia, mediastinitis, etc.).

### 4.2. Sample Collection

Patients’ venous blood was collected immediately before surgery in K3-EDTA tubes (Greiner Bio-One, Kremsmünster, Austria), after which a clinical blood analysis was performed. For gene expression analysis, erythrocytes were lysed and the leukocyte fraction was fixed in IntactRNA (Evrogen, Moscow, Russia) for −80 °C long-term storage. For serum protein concentration determination, patients’ venous blood was collected in gel tubes (Greiner Bio-One, Kremsmünster, Austria), centrifuged, and serum was obtained for subsequent −80 °C storage.

### 4.3. Clinical Blood Analysis

Peripheral blood lymphocytes, neutrophils, monocytes, eosinophils, and basophils counts were determined before surgical intervention using an automatic hematology analyzer Sysmex XN-1000 (Sysmex, Kobe, Japan). Based on blood analysis results, hematological indices were calculated: neutrophil-to-lymphocyte ratio NLR, monocyte-to-lymphocyte ratio MLR, and systemic inflammation index SIRI [40].

### 4.4. Real-Time PCR

RNA was isolated from leukocytes using the RNASolo kit (Evrogen, Moscow, Russia), and reverse transcription was performed using the MMLV RT Kit (Evrogen, Moscow, Russia). *HIF1A*, *EPAS1*, *IL1B*, and *IL6* mRNA expression was determined using qPCRmix-HS SYBR mixture (Evrogen, Moscow, Russia) and primers (Table S1) synthesized by Evrogen (Evrogen, Moscow, Russia) by real-time PCR relative to *GAPDH* expression level on a DTprime instrument (DNA-Technology, Moscow, Russia). Relative mRNA expression level was calculated by the ΔΔCq method [41]. The *HIF1A*/*EPAS1* expression ratio was also calculated.

For real-time PCR data normalization, *GAPDH* was selected as the reference gene. This choice was based on the results of our own preliminary studies evaluating the expression stability of the three most commonly used housekeeping genes—*ACTB*, *B2M*, and *GAPDH*—in whole blood samples from patients with aortic aneurysm (unpublished). Additional support for our selection comes from the findings of He et al. (2025), who identified *GAPDH* as one of the preferred reference genes for gene expression analysis in human peripheral blood following X-ray irradiation [42]. However, *GAPDH* instability was reported under certain conditions, including in peripheral blood mononuclear cells from patients with type 2 diabetes [43]. This approach is consistent with the Wardaszka et al. (2025), who demonstrated that reference gene stability is highly dependent on specific experimental conditions, cell types, and their activation state [44]. In their study on peripheral blood mononuclear cells cultured under normoxic and hypoxic conditions, *GAPDH* was not among the most stable genes, underscoring the importance of validating reference genes for each specific experimental model. Therefore, it was considered the use of *GAPDH* as a reference gene within our experimental framework to be justified.

### 4.5. ELISA

Serum HIF-1α, IL-1β, IL-6, IL-10, C-reactive protein, and endothelin-1 levels before surgery were determined by ELISA using Cloud-Clone Corp. (Houston, TX, USA) and FineTest (Wuhan, China) kits according to the manufacturer’s protocols. The color reaction development was assessed in an ANTHOS 2010 microplate analyzer (Anthos Labtec Instruments, Wals, Austria).

### 4.6. Statistical Methods

Statistical data processing was performed using Statistica 8.0 and GraphPad Prism 8.0 software. Outliers were identified using the ROUT test, and extreme values were excluded from further analysis if necessary. Indicators distribution type was determined by the Kolmogorov–Smirnov test. Since the data were not normally distributed, differences significance between indicators was determined using the nonparametric Mann–Whitney test. Data were expressed as median and interquartile range Me (LQ(25%); UQ(75%)). To assess the diagnostic performance of the studied parameters and to determine the optimal cut-off values, ROC curve analysis was performed using GraphPad Prism 8.0. The ROC curves were constructed by plotting sensitivity against 1-specificity for each possible cut-off value. The optimal cut-off point was determined as the value maximizing the Youden index (Youden index = sensitivity + specificity − 1), which corresponds to the point on the ROC curve farthest from the diagonal reference line. The AUC was calculated for each parameter. Differences were considered statistically significant at *p* < 0.05.

## 5. Conclusions

We performed a preoperative molecular and hematological predictors analysis of inflammatory complications after cardiac surgery. Patients with a complicated postoperative period had significantly lower total neutrophil count and NLR index, while eosinophil count, *HIF1A* expression level, and *HIF1A*/*EPAS1* ratio in peripheral blood leukocytes were significantly higher in comparison with patients without complications. At the same time, traditional risk factors (age, BMI, EuroSCORE II), perioperative parameters (CPB duration, MIT, total and intraoperative blood loss), and classical inflammation markers (leukocyte count and serum CRP, IL-1β, IL-6, IL-10 levels) did not differ between groups, indicating these parameters limited predictive value and pointing to hidden mechanisms search necessity determining individual complications predisposition. The *HIF1A*/*EPAS1* ratio, reflecting the initial hypoxic response imbalance, possesses the best diagnostic characteristics. These predictors preoperative levels assessment will allow timely patients stratification according to complications risk and preventive measures aimed at adverse outcomes incidence reduction and cardiac surgical treatment results improvement.

## Figures and Tables

**Table 1:** Clinical parameters of patients. Me (25–75%), *p*—statistical significance of differences, Mann–Whitney test.

Parameter	No Complications (*n* = 35)	with Complications (*n* = 16)	*p*-Value
Age, years	58.00 (43.00–66.00)	57.00 (43.75–64.25)	0.7
BMI	28.03 (25.36–31.58)	27.91 (25.78–32.10)	1.0
EuroSCORE II, %	3.28 (2.48–4.27)	3.26 (2.41–5.92)	0.8
CPB, min	110.50 (79.75–140.00)	125.50 (105.8–177.00)	0.1
MIT, min	87.00 (59.50–101.80)	90.00 (74.75–100.30)	0.5
Intraoperative blood loss, mL	700.0 (600.0–800.0)	650.0 (512.5–1000.0)	1.0
Total blood loss, mL	1050.0 (920.0–1250.0)	1075.0 (787.5–1515.0)	0.9
ICU stay length, days	1.00 (1.00–1.00)	1.50 (1.00–2.75)	0.01
Total hospitalization, days	10.00 (9.00–12.00)	11.50 (7.00–18.25)	0.4

**Table 2:** Leukocyte count, absolute lymphocyte, eosinophil, basophil, monocyte counts, MLR and SIRI indices, *EPAS1*, *IL1B*, and *IL6* expression levels in peripheral blood leukocytes, serum HIF-1α, IL-1β, IL-6, IL-10, C-reactive protein, and endothelin-1 concentrations in cardiac surgery patients, Me (25–75%), *p*—statistical significance of differences, Mann–Whitney test.

Parameter	No Complications (*n* = 35)	with Complications (*n* = 16)	*p*-Value
Leukocytes, ×10^9^/L	8.10 (6.90–9.80)	7.15 (6.10–9.15)	0.18
Lymphocytes, ×10^9^/L	1.87 (1.55–2.43)	2.13 (1.670–2.66)	0.21
Basophils, ×10^9^/L	0.04 (0.03–0.06)	0.05 (0.04–0.07)	0.14
Monocytes, ×10^9^/L	0.68 (0.55–0.77)	0.65 (0.57–0.80)	0.76
MLR	0.32 (0.27–0.46)	0.31 (0.22–0.40)	0.30
SIRI	1.85 (1.12–3.01)	1.18 (0.66–1.76)	0.06
*EPAS1*, expression level	0.11 (0.02–0.66)	0.07 (0.01–0.42)	0.46
*IL1B*, expression level	0.31 (0.08–0.44)	0.09 (0.05–0.33)	0.22
*IL6*, expression level	0.22 (0.08–0.53)	0.11 (0.04–0.32)	0.17
HIF-1α, ng/mL	2.32 (1.84–3.14)	2.34 (1.80–2.80)	0.60
IL-1β, pg/mL	0.28 (0.00–0.91)	0.68 (0.00–1.09)	0.28
IL-6, pg/mL	3.49 (1.65–7.87)	2.39 (1.96–4.90)	0.38
IL-10, pg/mL	3.86 (1.77–5.87)	2.36 (0.47–4.89)	0.14
C-reactive protein, IU/L	0.75 (0.36–3.62)	0.55 (0.20–0.95)	0.20
Endothelin-1, pg/mL	16.82 (12.68–49.62)	21.75 (12.64–61.76)	0.55

**Table 3:** Identified postoperative complications predictors diagnostic characteristics in cardiac surgery patients.

Parameter	AUC	Cut-Off Value	Diagnostic Sensitivity, %	Diagnostic Specificity, %	Likelihood Ratio
*HIF1A*/*EPAS1*	0.86	4.16	76.9	87.0	5.90
Neutrophils, ×10^9^/L	0.73	4.33	75.0	74.3	2.92
Eosinophils, ×10^9^/L	0.73	0.14	87.5	62.9	2.36
NLR	0.73	2.19	75.0	65.7	2.19
*HIF1A*, relative units	0.68	0.66	75.0	58.8	1.82

AUC—area under curve.

## Data Availability

The original contributions presented in this study are included in the article/Supplementary Material (Table S2). Further inquiries can be directed to the corresponding author.

## Supplementary Materials

The following supporting information can be downloaded at https://www.mdpi.com/article/10.3390/ijms27156616/s1.

## Abbreviations

The following abbreviations are used in this manuscript:

NLR	Neutrophil-to-Lymphocyte Ratio
ICU	Intensive Care Unit
HIF-1α	Hypoxia-Inducible Factor
CPB	Cardiopulmonary Bypass
MIT	Myocardial Ischemia Time
BMI	Body Mass Index
AUC	Area Under Curve
