# Supplementary material for: Predictive Value of Preoperative HIF1A and EPAS1 Expression Levels and Inflammatory Response Molecules for Assessing the Risk of Complications in Cardiac Surgery Patients
Source: Int J Mol Sci. 2026 Jul 24;27(15):6616. doi: 10.3390/ijms27156616 (PMC13465714; doi:10.3390/ijms27156616)
Supplement: Supplementary file 1 [file ijms-27-06616-s001.zip › ijms-4426705-supplementary/File S1. Informed Consent Form.pdf]

Informed Consent Form
for Participation in the Research Study FURG-2026-0033

I have understood the information provided to me regarding the research study FURG-2026-0033.
I have had sufficient time to make a decision. The researchers have explained any unclear information to me and have answered all my questions concerning participation in this research study.
I hereby confirm that I have been thoroughly informed about the risks and potential benefits of my participation in the research study FURG-2026-0033.
I voluntarily agree to participate in this study and to comply with the prescribed schedule of examinations.
I understand that participation in the study will include completing a questionnaire and providing a blood sample from the cubital vein for further analysis.
I authorize the physician-investigator and/or the research team to process my medical data for the purposes of the study, as well as to transfer such data in anonymized form.
I agree to provide access to my medical records to duly authorized officials, provided that the rules of personal data confidentiality are observed.
I have been provided with a signed and dated copy of the Participant Information Sheet and the informed consent form for participation in the study.
I have considered the possibility of using biological material and research results for scientific and diagnostic purposes:

I consent to the use of the biological material sample for scientific purposes in the study of similar diseases, subject to confidentiality requirements.	YES	NO
I consent to the use of the biological material sample for scientific purposes in the study of other diseases, subject to confidentiality requirements.	YES	NO
I consent to the long-term storage and use of the biological material sample for scientific purposes in future research, subject to confidentiality requirements.	YES	NO

An important question is who may receive the results of your research. Only you can determine whether you wish to know the results of the study and to whom you agree to have them disclosed. Please complete the sections below according to your preference:

I wish to receive the results of the study and obtain them personally (please indicate the method: by mail, by phone, in person)	YES	NO
I agree to the transfer of the results to an authorized representative<br>____________________<br>Full name of the authorized representative	YES	NO
Contact details of the authorized representative		

Based on the WMA Declaration on Ethical Considerations Regarding Health Databases and Biobanking, adopted by the 53rd WMA General Assembly in October 2002 and revised by the 67th WMA General Assembly in October 2016, we ask you to indicate the following:

I understand that I am donating my biological material for scientific research voluntarily and free of charge, and I do not expect any future payments for any commercial results and/or products obtained.

Yes ___________________________ No___________________________

I consent to the use of anonymized biological material and experimental research results for scientific purposes in similar studies conducted in the Russian Federation, as well as for subsequent publications in Russian and international peer-reviewed journals.

Yes ___________________________ No ____________________________

Your signature on this consent form signifies the following: You have understood all the information provided in Russian. This is a language you understand. You are satisfied with the explanations you have received regarding this study.

Patient's Full Name (please print clearly): ____

____

Patient's Signature ____

Date ____

Full Name of the Person Obtaining Consent (please print clearly): ____________________________________

____

Patient's Signature ____

Date ____
