# Supplementary material for: Predictive Value of Preoperative HIF1A and EPAS1 Expression Levels and Inflammatory Response Molecules for Assessing the Risk of Complications in Cardiac Surgery Patients
Source: Int J Mol Sci. 2026 Jul 24;27(15):6616. doi: 10.3390/ijms27156616 (PMC13465714; doi:10.3390/ijms27156616)
Supplement: Supplementary file 1 [file ijms-27-06616-s001.zip › ijms-4426705-supplementary/File S2. Patient Check list.pdf]

Patient Check list

Patient's Full Name (last, first, patronymic, if applicable)

____

Date of Birth (day, month, year)

____

No.	Question	Answer
1.	Do you feel well currently? (apart from the underlying disease)	Yes / No
2.	Have you ever had infectious diseases caused by human immunodeficiency virus (HIV infection), viral hepatitis B or C, syphilis, tuberculosis, or malaria?	Yes / No
3.	Have you ever had severe allergic reactions or bronchial asthma?	Yes / No
4.	Have you ever had seizures or diseases of the nervous system?	Yes / No
5.	Have you been in contact with persons with infectious diseases within the last 3 days?	Yes / No
6.	Have you had sexual contact with persons infected with human immunodeficiency virus (HIV), or with persons with viral hepatitis B or C, or syphilis?	Yes / No
7.	Have you stayed in areas where there is a threat of emergence and/or spread of mass infectious diseases or epidemics? If YES, please specify where: ______________________	Yes / No
8.	Have you ever used narcotic drugs or psychotropic substances?	Yes / No
9.	Are you currently taking or have you taken within the last 30 calendar days any medications, including antipyretics? If YES, please specify which: ______________________________	Yes / No
10.	Have you consumed alcohol within the last 48 hours?	Yes / No
11.	Are you registered for follow-up care or are you currently under the care of a physician? If YES, for what reason and in which medical facility? _________________________	Yes / No
12.	Have you undergone acupuncture, piercing, or tattooing within the last 120 calendar days?	Yes / No

Patient's Signature

____

Date (day, month, year)

____

Signature of Healthcare Professional

____
