# Supplementary material for: Predictive Value of Preoperative HIF1A and EPAS1 Expression Levels and Inflammatory Response Molecules for Assessing the Risk of Complications in Cardiac Surgery Patients
Source: Int J Mol Sci. 2026 Jul 24;27(15):6616. doi: 10.3390/ijms27156616 (PMC13465714; doi:10.3390/ijms27156616)
Supplement: Supplementary file 1 [file ijms-27-06616-s001.zip › ijms-4426705-supplementary/Table S1. Nucleotide sequences for real-time PCR.pdf]

Table S1. Nucleotide sequences for real-time PCR

| Gene | Direction | Sequence (5’→3’) |
|---|---|---|
| *GAPDH* | Forward | CGACCACTTTGTCAAGCTCA |
| | Reverse | AGGGGTCTACATGGCAACTG |
| *HIF1A* | Forward | GCAAGTCCTCAAAGCACAGT |
| | Reverse | TGGGTAGGAGATGGAGATGC |
| *EPAS1* | Forward | AGCACCCCTCTCTCTTCCATG |
| | Reverse | TTGGGTACATTTGCGCTCAGT |
| *IL1B* | Forward | CTGCTCTGGGATTCTCTTCAG |
| | Reverse | GAACCAGCATCTTCCTCAGC |
| *IL6* | Forward | GGTACATCCTCGACGGCAT |
| | Reverse | GGCATTTGTGGTTGGGTCA |
